# Patterns of perceived neighborhood environment and physical activity in adolescents: a latent class analysis

**DOI:** 10.1590/1980-549720250052

**Published:** 2025-11-21

**Authors:** Eduarda Cristina da Costa Silva, Juliana Maria da Penha Freire Silva, Gerfeson Mendonça, Alex Antonio Florindo, José Cazuza de Farias

**Affiliations:** I Physical Activity Epidemiology Research Group – João Pessoa (PB), Brazil.; II Joint Graduate Program in Physical Education – João Pessoa (PB), Brazil.; III Universidade de São Paulo, School of Arts, Sciences and Humanities – São Paulo (SP), Brazil.; IV Universidade Federal da Paraíba, Department of Physical Education – João Pessoa (PB), Brazil.

**Keywords:** Epidemiological studies, Built environment, Motor activity

## Abstract

**Objective:**

To identify patterns of characteristics of the perceived neighborhood environment and relate them to the types of physical activity practiced by adolescents.

**Methods:**

Observational epidemiological study with 1,066 adolescents (55.2% female, 10 to 13 years old) from João Pessoa, Paraíba, Brazil. Environmental characteristics were measured using a 16-item scale in three domains (places for practice, urban and traffic safety) and types of physical activity practice (recreation, sports, physical exercise and active commuting — minutes/week) by questionnaire. Latent Class Analysis (LCA) was applied to identify patterns of environmental characteristics and linear regression to relate them to the types of practice.

**Results:**

LCA identified four patterns of environmental characteristics: "not diverse and unsafe" (26.6%), "not diverse and safe" (13.6%), "diverse and unsafe" (30.8%), and "diverse and safe" (29.6%). Perceiving the environment as "diverse and safe" was positively and significantly associated with the time spent practicing recreational activities (β=42.16; 95%CI 4.35–79.97). The patterns of environmental characteristics were not associated with the time spent practicing sports, physical exercise, and active transportation.

**Conclusion:**

adolescents who perceived the neighborhood environment as "diverse and safe" had a longer time practicing recreational activities.

## INTRODUCTION

In recent years, the relationship between environmental factors and physical activity (PA) has received increasing attention^1,2^, particularly in light of existing theories and models addressing how characteristics of the physical and social environment may be associated with PA practices^3,4^. The settings and contexts in which individuals reside and interact have been shown to influence various domains of PA (leisure, active commuting, school/work, and home)^1,5^ as well as specific types of practice (recreational activities, sports, and physical exercise)^5–7^.

In general, the perception of availability and access to neighborhood facilities/equipment has been associated with participation in sports^7^ and leisure activities^1,2,5,6^, while greater urban and traffic safety has been linked to active commuting^5,7^. However, systematic reviews have reported null and/or inconsistent associations between environmental characteristics and PA among adolescents, regardless of the country in which the study was conducted, the measurement instruments employed, or the indicators used^1,2,8,9^. Such inconsistencies may be attributable to the varying operational concepts adopted to analyze both environmental characteristics and PA.

However, existing theories and models related to PA do not specify how environmental characteristics should be operationalized, nor do they explain the mechanisms through which these characteristics may be associated with PA. For analytical purposes, most studies have relied either on the individual use of each scale item^7^ or on the construction of scores by domain or as a total environmental score (sum of scale responses)^1,2,8,9^. Nevertheless, the environment comprises a combination of interrelated physical, social, and cultural characteristics that may influence PA in diverse ways^10,11^. Among adolescents, PA practice is marked by interest in specific types of activity^12^, which may, in turn, require different environmental characteristics^7^.

In this context, traditional data analysis approaches — such as generating overall scores, domain scores, or analyzing isolated items — may be insufficient to explain the relationships between environmental characteristics and the types of PA practiced^1,2,8,9^. Within this theoretical framework, the present study acknowledges that analyzing environmental variables in isolation may fail to capture the complexity of adolescents’ lived contexts. Latent Class Analysis (LCA) provides a more integrated approach by identifying patterns of environmental perception across multiple interdependent dimensions. This method makes it possible to distinguish subgroups of adolescents with similar response patterns, combining perceived environmental characteristics from different domains, such as the availability of PA facilities and urban and traffic safety^13–15^.

Most studies examining patterns of environmental characteristics and their relationship with PA have been conducted with adolescents in European and North American countries^16–18^. The associations observed in these studies may not necessarily apply to Brazil, given local social, cultural, and public policy factors that uniquely influence PA practices, particularly when compared to upper-middle-income countries. In Brazil, two studies involving adolescents and using the LCA have been identified^19,20^, and only one specifically aimed to identify patterns based on individual responses to each attribute of the perceived physical and social environment^19^.

Castro et al.^19^ identified three environmental patterns: adolescents and young adults (15 to 24 years old, from the city of Camaçari, Bahia) who perceived their neighborhood as "urbanized and PA-friendly" were 72% more likely to engage in leisure-time PA compared to those who perceived it as "unsafe and unsociable." However, this study focused on older adolescents and young adults, who not only have greater autonomy to navigate their neighborhoods but also exhibit different PA preferences^12,21^. From this perspective, the present study aimed to identify patterns of perceived neighborhood environmental characteristics and examine their relationship with the types of PA practiced among adolescents.

## METHODS

This observational, cross-sectional epidemiological study utilized data from the first wave (2014) of the Longitudinal Study on Sedentary Behavior, Physical Activity, Eating Habits, and Health (LONCAAFS). The study was approved by the Human Research Ethics Committee of the Health Sciences Center of Universidade Federal da Paraíba (No. 0240/13), and participation was authorized by the students’ parents/guardians, as well as by the school administration.

The target population comprised adolescents of both genders enrolled in the sixth grade of public schools (state and municipal) in João Pessoa, Paraíba. The sample size was calculated for a prevalence study, considering a target population of 9,520 adolescents, an outcome prevalence of 50% (chosen to maximize the required sample size for a given margin of error), a 95% confidence interval, an acceptable error of 4 percentage points, and a design effect (deff) of 2, resulting in a sample of 950 adolescents. To account for potential losses and refusals, the sample was increased by 40%, yielding a total of 1,582 adolescents.

Twenty-eight schools (14 municipal and 14 state) were systematically selected, distributed proportionally by geographic region (north, south, east, and west) and by the number of sixth-grade students enrolled. At each selected school, all sixth-grade students in 2014 were invited to participate. Data collection was conducted between February and December of each year (2014–2017) by a trained team following a standardized protocol. A questionnaire was administered via face-to-face interviews during the students’ class periods, and anthropometric measurements were also obtained.

Adolescents outside the target age range (<10 and >13 years), those with disabilities preventing them from completing the questionnaire, and those with missing data for one or more variables of interest were excluded from the analyses.

Environmental characteristics related to PA were assessed using a 16-item scale organized into three domains:

- presence of facilities for PA (8 items);
- urban safety (4 items);
- traffic safety (4 items)^22^.

To assess the presence of PA venues, adolescents reported whether such venues existed in their neighborhood (yes or no) and the travel time from their homes to these venues (1 to 5 minutes, 6 to 10 minutes, 11 to 20 minutes, or >20 minutes). Urban safety and traffic safety each comprised four items. Adolescents indicated their perceptions (yes or no) regarding "*fear of being in open spaces, on the streets during the day and at night, and traffic flow*." For analysis, responses in domain I were recoded as "yes" or "no" to indicate the presence of a venue in the neighborhood, while responses in Domains II and III were analyzed as originally measured.

PA was assessed using the Physical Activity Questionnaire for Adolescents (PAQA)^23^, which includes a list of 19 moderate- to vigorous-intensity activities and active transportation, with space for adolescents to add up to two additional activities. Participants reported whether they engaged in each activity (yes or no), its frequency (days/week), and its duration (minutes/day) during the week preceding data collection. Only activities lasting at least 10 minutes were considered. For analysis, weekly minutes were calculated by summing the products of frequency and duration for each activity, which were then grouped into the following categories: recreation (dancing, cycling, and playing); sports (basketball, handball, volleyball, beach volleyball, swimming, soccer, beach soccer, futsal, wrestling, rhythmic gymnastics, skateboarding, surfing, track and field, and tennis); physical exercise (aerobic gymnastics, weight training, walking, and running); and active travel (walking/cycling to school and other destinations).

The sociodemographic variables included in this study were gender; age in complete years; mother's education; class shift; skin color; economic class^24^; time of residence in the neighborhood; and Body Mass Index (BMI) — calculated from measured weight and height. Nutritional status was classified according to the World Health Organization (WHO) criteria^25^: underweight (<-2 standard deviations [sd]), normal weight (>-2 sd and <+1 sd), overweight (>+1sd), and obesity (>+2 sd).

Descriptive statistics, including means, standard deviations, and frequency distributions, were used to summarize the data. The χ^2^ test and Mann-Whitney U test were employed to compare sociodemographic characteristics and types of PA between adolescents included in and excluded from the analyses. Crude binary logistic regression was used to determine whether the pattern of missing data was completely random, random, or non-random^26^.

LCA was employed to identify item response patterns (no=0, yes=1) for the measured environmental characteristics (Supplementary Material 1). To evaluate model fit and determine the optimal number of classes, several criteria were applied: lowest values of the Akaike Information Criterion (AIC), Bayesian Information Criterion (BIC), and adjusted BIC; entropy values closest to one; and the Lo-Mendell-Rubin Test (LMR), Bootstrap Likelihood Ratio Test (BLRT), and Likelihood Ratio Test (LRT). These tests assess whether a model with an additional class (*k* +1) provides a significantly better fit than a model with fewer classes (*k*). The model with fewer classes was rejected when the comparison yielded a p-value <0.05.

Crude and adjusted linear regression analyses were conducted to examine the relationship between environmental patterns (independent variable) and types of PA (dependent variable). The following confounding factors were considered: gender (male=0, female=1), age (calculated from the difference between birth date and data collection date, categorized as 10-11 years=0 and 12-13 years=1), economic class (A/B=0, C/D/E=1), mother's education (complete elementary=0, incomplete high school=1, complete high school or higher=2), skin color (mixed race, black, white, Asian, and indigenous, and recategorized as: white=0, non-white=1), class shift (morning=0, afternoon=1), time of residence in the neighborhood (<1 year=0 and ≥1 year=1), and nutritional status (underweight/normal weight=0, overweight/obese=1). Forward selection was used for variable inclusion in the adjusted model, retaining those that improved model fit. Model diagnostics included the variance inflation factor (VIF), with values <5 indicating absence of multicollinearity; the Shapiro-Wilk test to assess residual normality; and the Cook-Weisberg test to assess residual homoscedasticity (with White's robust correction applied in the presence of heteroscedasticity).

Descriptive and association analyses were conducted using Stata 13.0, while LCA was performed in M-plus 7.0. A significance level of p<0.05 was adopted.

## RESULTS

Of the 1,582 adolescents invited, 1,475 participated in the study. After accounting for refusals and losses (n=107), the final sample comprised 1,066 adolescents. Significant differences were observed between included and excluded adolescents for gender (p=0.004) and economic class (p=0.008) (Table 1). The missing data were classified as random (data not shown).

**Table 1:** Comparison of sociodemographic characteristics, body mass index (BMI), and physical activity between adolescents included and excluded from the analyses. João Pessoa, Paraíba, 2014.

Characteristics		Adolescents included in the analyses (n=1,066)	Adolescents excluded from the analyses (n=409)	Valor p†
		n (%)	n (%)	
Gender				
	Male	477 (44.7)	217 (53.1)	0.004
	Female	589 (55.2)	192 (46.9)	
Age (years)				
	10 to 11	655 (61.4)	176 (59.3)	0.495
	12 to 13	411 (38.6)	121 (40.7)	
Skin color				
	White	211 (19.8)	73 (17.9)	0.410
	Non-white	853 (80.2)	334 (82.1)	
School shift				
	Morning	475 (44.6)	179 (43.7)	0.784
	Afternoon	591 (55.4)	230 (56.2)	
Mother's education				
	Incomplete elementary	422 (39.6)	71 (47.9)	0.059
	Incomplete high school	303 (28.4)	43 (29.1)	
	Complete high school or higher	341 (31.9)	34 (22.9)	
Economic status				
	A/B (higher)	452 (42.4)	68 (32.5)	0.008
	C/D/E (medium and lower)	614 (57.6)	141 (67.5)	
Nutritional status				
	Underweight/normal weight	717 (67.3)	275 (69.6)	0.391
	Overweight/obesity	349 (32.7)	120 (30.4)	
		**Mean (standard deviation)**	**Mean (standard deviation)**	**Valor p** *
Physical activity (min/week)				
	Recreation	168.2 (231.5)	158.6 (217.5)	0.153
	Sports	233.4 (293.3)	228.7 (286.1)	0.353
	Physical exercise	42.6 (110.5)	42.1 (115.2)	0.121
	Active commuting	133.1 (134.5)	137.8 (150.4)	0.977

† p-value from the χ^2^ test;

* p-value from the Mann-Whitney U test; min/week=minutes per week.

**Figure 1:**
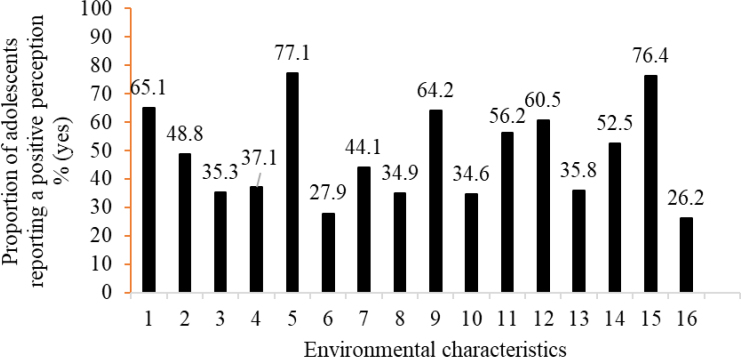
Proportion of adolescents reporting a positive perception of environmental characteristics for physical activity in João Pessoa, Paraíba, in 2014.

The majority of adolescents were female (52.2%), aged 10-11 years (61.4%), and belonged to economic class C/D/E (57.6%) (Table 1). Regarding perceived environmental characteristics, a high proportion of adolescents reported the presence of "squares" (77%; places for PA); "not being afraid to be in open spaces" (64.2%; urban safety); and "safety when crossing streets" (76.4%; traffic safety) (Figure 1).

1: gyms or martial arts training centers; 2: outdoor sports courts; 3: indoor multi-sport gymnasium; 4: walking/running tracks; 5: squares/plazas; 6: parks/green areas; 7: playgrounds; 8: skate/roller skating tracks; 9: not afraid to stay in open areas; 10: not afraid to stay in open areas with friends; 11: not afraid to walk on the streets (day); 12: not afraid to walk on the streets (night); 13: no heavy car/motorcycle traffic; 14: no speeding drivers; 15: safe street crossings; 16: low incidence of robberies/thefts.

**Figure 2:**
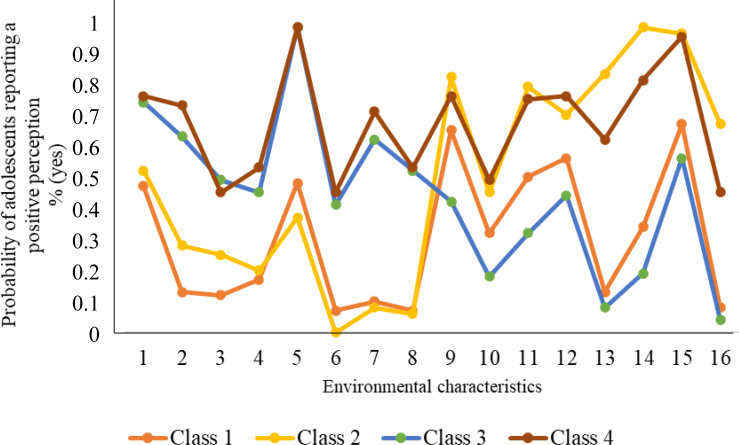
Latent classes of neighborhood environmental patterns for physical activity among adolescents. João Pessoa, Paraíba, 2014.

Five models were evaluated sequentially (*k + 1* classes), and based on model fit indicators, the four-class model was identified as the best fit (Table 2). The classes were labeled as follows: "non-diversified and insecure" (Class 1, n=277 — 26%), "non-diversified and secure" (Class 2, n=145 — 13.6%), "diversified and insecure" (Class 3, n=328 — 30.8%), and "diversified and secure" (Class 4, n=316 — 29.6%) (Figure 2).

Class 1: n=277 (26%) — non-diverse–unsafe; Class 2: n=145 (13.6%) — non-diverse–safe; Class 3: n=328 (30.8%) — diverse–unsafe; and Class 4: n=316 (29.6%) — diverse–safe; X-axis: represents the items composing the scale measuring environmental characteristics; Y-axis: represents the probability that the adolescent perceives the presence of the item in the neighborhood positively ("yes"); 1: gyms or martial arts training centers; 2: outdoor sports courts; 3: indoor multi-sport gymnasium; 4: walking/running tracks; 5: squares/plazas; 6: parks/green areas; 7: playgrounds; 8: skate/roller skating tracks; 9: not afraid to stay in open areas; 10: not afraid to stay in open areas with friends; 11: not afraid to walk on the streets (day); 12: not afraid to walk on the streets (night); 13: no heavy car/motorcycle traffic; 14: no speeding drivers; 15: safe street crossings; 16: low incidence of robberies/thefts.

**Table 2:** Model fit parameters for the formation of latent classes based on environmental characteristics for physical activity among adolescents in João Pessoa, Paraíba, in 2014.

Model parameters		Latent classes					
Number of latent classes			1	2	3	4	5
Log-Likelihood			-10,906.4	-10,450.1	-10,151.7	-10,032.2	-9,982.3
AIC^a^			21,844.7	20,966.9	20,403.5	20,198.4	20,132.7
BIC^a^			21,924.2	21,131.0	20,652.1	20,531.5	20,550.3
BIC – Adjusted			21,873.4	21,526.1	20,493.3	20,318.7	20,283.5
VLMR (p-value)			-	-10,906 (<0.001)	-10,450.4 (0.003)	-10,151.7 (<0.001)	-10,032.2 (0.376)
LMR (p-value)			-	904.1 (<0.001)	592.4 (0.003)	237.1 (<0.001)	98.8 (0.379)
BRLT (p-value)			-	-10,906.4 (<0.001)	-10,450.5 (<0.001)	-10,151.7 (<0.001)	-10,032.2 (<0.001)
Entropy^b^			1.000	0.738	0.736	0.737	0.721
Classes		n (%)	Likelihood				
			1	2	3	4	5
1 class1		1,066 (100)	1.000				
2 classes							
	class 1	645 (60.4)	0.924	0.076			
	class 2	421 (39.5)	0.079	0.921			
3 classes							
	class 1	331 (31)	0.866	0.086	0.048		
	class 2	380 (35.6)	0.065	0.881	0.054		
	class 3	355 (33.2)	0.053	0.053	0.894		
4 classes							
	class 1	145 (13.6)	0.865	0.070	0.064	0.002	
	class 2	277 (26.0)	0.048	0.858	0.019	0.074	
	class 3	315 (29.6)	0.039	0.027	0.839	0.095	
	class 4	328 (30.8)	0.001	0.069	0.072	0.858	
5 classes							
	class 1	267 (25.1)	0.829	0.036	0.076	0.046	0.013
	class 2	141 (13.2)	0.072	0.861	0.003	0.002	0.062
	class 3	167 (15.6)	0.108	0.008	0.736	0.079	0.068
	class 4	228 (21.4)	0.034	0.001	0.077	0.832	0.057
	class 5	263 (24.7)	0.012	0.053	0.039	0.054	0.841

AIC: Akaike Information Criterion; BIC: Bayesian Information Criterion; VLMR: Vuong-Lo-Mendell-Rubin Likelihood Ratio Test; LMR: Lo-Mendell-Rubin Adjusted; BRLT: Bootstrap Likelihood Ratio Test;

a Lower values indicate better model fit;

b Measure of classification uncertainty ranging from 0 to 1, with higher values reflecting better model fit.

In the crude analysis, perceiving the environment as "diverse and safe" was positively and significantly associated with recreational activities (β=42.08; 95%CI: 5.09–71.64) and sports (β=63.07; 95%CI: 16.27–109.87). In the adjusted analysis (Table 3), the association with sports lost statistical significance, while the relationship with recreational activities remained significant (β=42.16; 95%CI: 4.35–79.97).

**Table 3:** Crude and adjusted linear regression for the association between perceived neighborhood environmental patterns and types of physical activity among adolescents in João Pessoa, Paraíba, in 2014.

Characteristics	Types of practice (minutes/week)							
	Recreation		Sports		Physical exercise		Active commuting	
	Crude model	Adjusted model	Crude model	Adjusted model	Crude model	Adjusted model	Crude model	Adjusted model
	β (95%CI)	β (95%CI)	β (95%CI)	β (95%CI)	β (95%CI)	β (95%CI)	β (95%CI)	β (95%CI)
Class 1	1	1	1	1	1	1	1	1
Class 2	43.41 (-4.02–90.86)	41.10 (-7.21–89.42)	40.65 (-19.36–100.67)	-25.89 (-82.99–31.19)	6.66 (-16.02–29.35)	3.68 (-19.27–26.63)	5.41 (-22.14–32.97)	4.99 (-22.87–32.85)
Class 3	34.38 (-2.86–71.63)	33.90 (-3.37–71.18)	12.48 (-34.64–59.61)	14.34 (-29.70–58.40)	10.07 (-7.73–27.89)	9.52 (-8.18–27.24)	18.34 (-3.29–39.98)	17.73 (-3.76–39.24)
Class 4	42.08 (5.09–71.64)	42.16 (4.35–79.97)	63.07 (16.27–109.87)	7.24 (-37.43–51.92)	10.69 (-6.99–28.38)	9.66 (-8.30–27.62)	-5.23 (-26.72–16.25)	-1.84 (-23.65–19.95)

β: beta coefficient; 95%CI: 95% confidence interval; Adjusted model for gender, age, economic class, mother's education, skin color, nutritional status, and school shift; Class 1: "non-diverse and unsafe"; Class 2: "non-diverse and safe"; Class 3: "diverse and unsafe"; Class 4: "diverse and safe". VIF values in all models were <2, indicating no multicollinearity; the Cook-Weisberg test showed p<0.001, indicating heteroscedasticity, and White's robust correction was applied.

### Data availability statement:

The dataset supporting the findings of this study is not publicly available.

## DISCUSSION

This study identified four distinct patterns of perceived environmental characteristics ("non-diverse and unsafe," "non-diverse and safe," "diverse and unsafe," and "diverse and safe") in relation to adolescents’ PA. Among these, the "diverse and safe" pattern was associated with recreational activities.

The hypothesis that adolescents perceive environmental characteristics heterogeneously — particularly when multiple domains, such as the availability of PA facilities and urban and traffic safety, are combined — was confirmed. This finding reflects how adolescents perceive and interpret their environment in real-life contexts. Previous studies conducted in Brazil have also identified up to four distinct environmental patterns, taking into account adolescents’ perceptions of PA facility availability and safety-related aspects^19,20^.

Only Castro et al.^19^ investigated patterns ("urbanized and PA-friendly neighborhood," "safe and sociable neighborhood," and "unsafe and unsociable") based on individual responses to each measured environmental characteristic. Of these patterns, the "urbanized and PA-friendly neighborhood" was associated with higher odds of engaging in leisure-time PA (OR=1.72; 95%CI: 1.29–2.29). It is important to note that the participants in that study were older adolescents and young adults^19^, who generally have greater autonomy to navigate their neighborhoods and specific preferences for certain types of activities, in contrast to younger adolescents, who are more dependent on their parents and tend to prefer other types of activities.

In this study, adolescents who perceived their neighborhood as "diverse and safe" spent more time engaging in recreational activities than those who perceived it as "non-diverse and unsafe." This association may reflect a subgroup of adolescents whose parents and/or guardians have higher socioeconomic status and/or greater knowledge about PA. Consequently, these adolescents may reside in neighborhoods with recreational facilities, equipment, and adequate urban and traffic safety, facilitating greater participation in recreational activities.

Another explanation for this finding may relate to the inherent characteristics of recreational activities (*e.g.*, such as cycling and playing), which generally do not require prior planning, fixed schedules, formal rules, specialized facilities, and/or specific equipment. Consequently, neighborhoods offering greater diversity of PA locations and low crime rates may be more conducive to recreational activities, encouraging adolescents to engage in these activities during their leisure time.

The absence of an association between the identified environmental patterns and physical exercise or sports in this study may be attributable to the characteristics and requirements of these types of PA, which typically demand specific locations, spaces, equipment, and materials. Although previous studies involving adolescents of a similar age have reported positive associations between environmental characteristics and engagement in physical exercise^27^ and sports^7^, this relationship was not observed in the present study. This discrepancy may be related to the PA questionnaire used, which did not capture the locations where adolescents performed the reported activities.

Although physical exercise can be performed in a variety of settings, such as outdoors or in gyms/fighting centers, it often requires guidance on proper practice and access to specific equipment. This may pose a barrier for adolescents from lower socioeconomic backgrounds, as the majority of participants in this study (57.6%) belonged to middle- or lower-income classes. Regarding the lack of association between environmental patterns and sports activities, the school environment and physical education classes may play a more influential role for adolescents who lack access to neighborhood spaces, materials, or equipment. Schools may also serve as the primary venues for sports participation, providing opportunities to engage in activities such as volleyball, futsal, and other team sports alongside peers.

This study also found no association between environmental patterns and active commuting. This may be because active commuting is often undertaken out of necessity rather than choice, due to limited transportation options for traveling between home, school, friends’ houses, or other destinations^28^. Previous studies have shown that active commuting to school is associated with lower family income^29^, attendance at public schools^30^, and the absence of household transportation — such as a car^31^.

Although a validated scale was used to assess environmental characteristics^22^, it is possible that other unmeasured factors may have been more predictive of the types of PA practiced by adolescents in this study. Research using shorter environmental scales^19^ has reported less diversity in the associations between identified patterns and PA compared with studies employing scales with a larger number of domains and items^20^. However, these studies measured total PA, leisure-time PA^19^, or PA by intensity (light, moderate, vigorous)^20^, which may not reflect adolescents’ specific interests and preferences. This limitation could reduce the ability to assess how perceptions of a set of environmental characteristics relate to distinct types of PA.

The findings of this study highlight the potential of adopting an alternative approach to analyzing environmental characteristics, demonstrating that perceptions of specific attributes, when considered together, are associated with particular types of PA. This insight may inform the development of interventions aimed at modifying either the physical environment of neighborhoods or adolescents’ perceptions of their surroundings. Future studies on environmental patterns and PA are encouraged to include additional environmental characteristics to further examine their relationship with different types of PA among adolescents.

This study has several limitations. The questionnaire used to assess adolescents’ PA did not include information on the location of the activities (*e.g*., at school or in the neighborhood). Given that most participants were from middle/lower-income backgrounds, it is possible that many reported activities were performed at school or during physical education classes. Another limitation relates to the environmental scale, as the relatively small number of items may not have captured neighborhood characteristics that are more relevant to the types of PA analyzed, potentially influencing the number of associations identified.

Despite these limitations, the study employed validated instruments to assess environmental characteristics^22^ and PA^23^, administered by a trained team following a standardized data collection protocol. In addition, a robust statistical analysis was conducted, enabling the identification of potential combinations of attributes across different domains of the perceived environment and their associations with types of PA among adolescents.

In conclusion, characteristics across different environmental domains, including the availability of PA facilities and urban and traffic safety, are interrelated. When considered together, adolescents exhibited heterogeneous perception patterns, indicating that neighborhoods do not affect all individuals equally. Adolescents who perceived their neighborhood environment as diverse and safe engaged more frequently in recreational activities.
