# Supplementary material for: Patterns of perceived neighborhood environment and physical activity in adolescents: a latent class analysis
Source: Rev Bras Epidemiol. 2025 Nov 21;28:e250052. doi: 10.1590/1980-549720250052 (PMC12643389; doi:10.1590/1980-549720250052)
Supplement: Material Suplementar 1 [file 1980-5497-rbepid-28-e250052-Suppl01.pdf]

**Padrões das características do ambiente percebido do bairro e atividade física em adolescentes: uma Análise de Classes Latentes**

Material Suplementar 1 – Instruções realizadas no modelo final para a Análise de Classes Latentes no MPLUS

```
Mplus VERSION 7
MUTHEN & MUTHEN
11/11/2024   6:35 PM

INPUT INSTRUCTIONS

  TITLE: Análise de Classe Latente

  DATA:
  FILE IS padroesdoambiente.dat;

  VARIABLE:
  NAMES ARE id amb1 amb4 amb5 amb7 amb9 amb10 amb11
  amb13 sba sbb sbd sbg sbh sbi sbj sbk;

  USEVARIABLES ARE amb1 amb4 amb5 amb7 amb9 amb10 amb11
  amb13 sba sbb sbd sbg sbh sbi sbj sbk;

  IDVARIABLE = id;

  CATEGORICAL ARE amb1 amb4 amb5 amb7 amb9 amb10 amb11
  amb13 sba sbb sbd sbg sbh sbi sbj sbk;


  CLASSES = c(4);

  TYPE = MIXTURE;
  ESTIMATOR = mlr;
  STARTS = 60 30;
  STITERATIONS = 30;
  PROCESSORS = 8 (STARTS);

  OUTPUT: TECH1 TECH8 TECH10 TECH14;

  PLOT:
  SERIES = amb1(1) amb4(2) amb5(3) amb7(4) amb9(5) amb10(6)
  amb11(7) amb13(8) sba(9) sbb(10) sbd(11) sbg(12) sbh(13)
  sbi(14) sbj(15) sbk(16);

  TYPE = PLOT3;

  SAVEDATA:
  FILE IS probabilidades.csv;
  FORMAT IS free;
```
