# Supplementary material for: Patterns of perceived neighborhood environment and physical activity in adolescents: a latent class analysis
Source: Rev Bras Epidemiol. 2025 Nov 21;28:e250052. doi: 10.1590/1980-549720250052 (PMC12643389; doi:10.1590/1980-549720250052)
Supplement: Translation

# Translation: Padrões das características do ambiente percebido do bairro e atividade física em adolescentes: uma análise de classes latentes

**DOI:** 10.1590/1980-549720250052.2

**Authors:** Eduarda Cristina da Costa Silva, Juliana Maria da Penha Freire Silva, Gerfeson Mendonça, Alex Antonio Florindo, José Cazuza de Farias

**Affiliations:** I Grupo de Estudos e Pesquisas em Epidemiologia da Atividade Física – João Pessoa (PB), Brasil.; II Programa Associado de Pós-Graduação em Educação Física – João Pessoa (PB), Brasil.; III Universidade de São Paulo, Escola de Artes, Ciências e Humanidades – São Paulo (SP), Brasil.; IV Universidade Federal da Paraíba, Departamento de Educação Física – João Pessoa (PB), Brasil.

**Keywords:** Estudos epidemiológicos, Ambiente construído, Atividade motora

## Abstract

**Objetivo:**

Identificar os padrões das características do ambiente percebido do bairro e relacioná-los com os tipos de prática de atividade física (AF) em adolescentes.

**Métodos:**

Estudo epidemiológico observacional, com 1.066 adolescentes (55,2% do sexo feminino, de 10 a 13 anos de idade) de João Pessoa, Paraíba, Brasil. As características do ambiente foram mensuradas por meio de escala com 16 itens em 3 domínios (locais para AF, segurança urbana e no trânsito) e os tipos de prática de atividade física (recreação, esportes, exercícios físicos e deslocamento ativo — minutos/semana), por questionário. A Análise de Classes Latentes (ACL) foi aplicada para identificar os padrões das características do ambiente e a regressão linear para relacioná-los com os tipos de prática.

**Resultados:**

A ACL identificou quatro padrões das características do ambiente: "não diversificado e inseguro" (26,6%), "não diversificado e seguro" (13,6%), "diversificado e inseguro" (30,8%), e "diversificado e seguro" (29,6%). Perceber o ambiente como "diversificado e seguro" associou-se de forma positiva e significativa ao tempo de prática de atividades recreativas (β=42,16; IC95% 4,35–79,97). Os padrões das características do ambiente não se associaram ao tempo de prática de esportes, exercícios físicos e deslocamento ativo.

**Conclusão:**

Adolescentes que percebiam o ambiente do bairro "diversificado e seguro" apresentaram maior tempo de prática em atividades recreativas.

## INTRODUÇÃO

Nos últimos anos, a relação entre os fatores ambientais e a atividade física (AF) tem recebido cada vez mais atenção^1,2^, considerando as teorias e os modelos existentes sobre o quanto as características do ambiente físico e social podem estar relacionadas à prática da AF^3,4^. Os locais e os contextos onde as pessoas residem e interagem têm exercido influência nos diferentes domínios da AF (lazer, deslocamento ativo, escola/trabalho e lar)^1,5^ e nos tipos de prática (atividades recreativas, esportes e exercícios físicos)^5–7^.

De maneira geral, perceber a disponibilidade e o acesso a instalações/equipamentos no bairro têm sido associados a prática de esportes^7^ e de lazer^1,2,5,6^ e maior segurança urbana e no trânsito ao deslocamento ativo^5,7^. No entanto, revisões sistemáticas identificaram associações nulas e/ou inconsistentes entre as características do ambiente e a AF em adolescentes, independentemente do país onde o estudo foi realizado, do instrumento de mensuração e dos indicadores utilizados^1,2,8,9^. Isso pode ser decorrente dos diferentes conceitos operacionais adotados para analisar tanto as características do ambiente quanto os da AF.

No entanto, as teorias e os modelos relacionados à AF não descrevem como as características do ambiente deveriam ser operacionalizadas, nem os mecanismos pelos quais podem estar associadas com a AF. A maioria dos estudos têm considerado para fins de análise o uso individual de cada item da escala^7^ ou a formação de escores por domínio ou total do ambiente (somatório das respostas da escala)^1,2,8,9^. No entanto, o ambiente é composto por uma combinação de características físicas, sociais e culturais, que estão inter-relacionadas e podem influenciar de diferentes formas a AF^10,11^. Em adolescentes, a prática de AF é caracterizada pelo interesse nos tipos^12^, e isso pode demandar características variadas do ambiente^7^.

Nesse contexto, abordagens tradicionais na análise dos dados — como a formação de escore geral, por domínios ou itens isolados – podem não ser adequadas para explicar as relações entre as características do ambiente e os tipos de AF praticadas^1,2,8,9^. Ao adotar esse arcabouço teórico, o presente estudo reconhece que a análise isolada de variáveis ambientais pode não capturar a complexidade dos contextos vivenciados pelos adolescentes. Assim, a aplicação da Análise de Classes Latentes (ACL) possibilita uma abordagem mais integrada, identificando padrões de percepção ambiental com base em múltiplas dimensões interdependentes. Essa abordagem permite identificar diferentes subgrupos de adolescentes que apresentam um padrão de resposta semelhante, combinando características do ambiente percebido de diversos domínios, como disponibilidade de locais para AF e segurança urbana e no trânsito^13–15^.

A maioria dos estudos que investigaram padrões das características do ambiente e a sua relação com a AF foram realizados com adolescentes de países da Europa e América do Norte^16-18^. É possível que as associações identificadas nesses estudos possam não ser encontradas no Brasil, devido às características locais, sociais, culturais e de políticas públicas que influenciam a prática de AF específicas de cada região, principalmente quando comparadas aos países de renda média alta. No Brasil, foram identificados dois estudos com adolescentes que utilizaram a ACL^19,20^, e apenas um buscou identificar os padrões com base nas respostas individuais de cada atributo do ambiente físico e social percebido^19^.

Castro et al.^19^ identificaram três padrões de ambiente; os adolescentes e adultos (15 a 24 anos de idade, da cidade de Camaçari, Bahia) que percebiam o bairro como "vizinhança urbanizada e favorável à AF" apresentaram 72% mais chances de realizar a AF no lazer, comparados aos que percebiam a "vizinhança insegura e pouco sociável". No entanto, esse estudo foi realizado com adolescentes mais velhos e adultos mais jovens, que, além de terem maior autonomia para se deslocar no bairro, têm outras preferências de prática de AF^12,21^. Nessa perspectiva, o presente estudo teve como objetivo identificar os padrões das características do ambiente percebido do bairro e relacioná-los com os tipos de prática de AF em adolescentes.

## MÉTODOS

Trata-se de um estudo epidemiológico observacional, do tipo transversal, que utilizou dados da primeira onda (2014) do Estudo Longitudinal sobre Comportamento Sedentário, Atividade Física, Hábitos Alimentares e Saúde (LONCAAFS). Este estudo foi aprovado pelo Comitê de Ética em Pesquisa com Seres Humanos do Centro de Ciências da Saúde da Universidade Federal da Paraíba (n.° 0240/13) e os participantes da pesquisa, autorizados pelos pais/responsáveis e a direção da escola.

A população-alvo foi composta de adolescentes de ambos os sexos, matriculados nos sextos anos do ensino fundamental de escolas públicas (estaduais e municipais) de João Pessoa, Paraíba. O cálculo de tamanho da amostra foi realizado com base em um estudo de prevalência: tamanho da população-alvo igual a 9.520 adolescentes; prevalência do desfecho de 50% (foi selecionada a prevalência de 50% por produzir o maior número de participantes para a mesma margem de erro); intervalo de confiança de 95%; erro aceitável de 4 pontos percentuais; efeito de delineamento (*deff*) de 2, resultando uma amostra de 950 adolescentes. Para compensar possíveis perdas e recusas, houve um acréscimo de 40%, totalizando uma amostra de 1.582 adolescentes.

Foram selecionadas sistematicamente 28 escolas (14 municipais e 14 estaduais), distribuídas proporcionalmente por região geográfica (norte, sul, leste e oeste) e pelo número de alunos matriculados nos sextos anos. Nas escolas selecionadas, todos os escolares dos sextos anos em 2014 foram convidados a participar do estudo. A coleta de dados foi realizada entre os meses de fevereiro e dezembro de cada ano (2014-2017) por uma equipe treinada, seguindo um protocolo padronizado e aplicando um questionário, por meio de entrevista face a face, na escola e no turno que o escolar estudava — além de realizadas as medidas antropométricas.

Foram excluídos das análises do presente estudo os adolescentes que estavam fora da faixa etária de interesse (<10 e >13 anos de idade), com algum tipo de deficiência que os impossibilitavam de responder ao questionário e com dados ausentes (*missing*) para uma ou mais variáveis de interesse deste estudo.

As características do ambiente para a AF foram mensuradas por meio de uma escala composta por 16 itens, em 3 domínios:

- presença de locais para a AF (8 itens);
- segurança urbana (4 itens);
- segurança no trânsito (4 itens)^22^.

Para mensurar a presença de locais para a prática de AF, o adolescente informava sobre a existência de locais no bairro (sim versus não) e o tempo de deslocamento da sua residência até esses locais (1 a 5 minutos, 6 a 10 minutos, 11 a 20 minutos, >20 minutos). A segurança urbana e a segurança no trânsito foram compostas por quatro itens cada um. O adolescente informava a sua percepção (não versus sim) sobre itens relacionados *"ao medo de ficar em locais abertos, nas ruas durante o dia e à noite, e fluxo de veículos"*. Para fins de análise, as respostas em cada item do domínio I foram recategorizadas em "sim versus não" para o local no bairro, enquanto as dos domínios II e III foram analisadas da forma como foram mensuradas.

A AF foi mensurada por meio do Questionário de Atividade Física para Adolescentes (QAFA)^23^, composto por uma lista de 19 atividades de intensidade moderada à vigorosa e o deslocamento ativo, com possibilidade de adolescente acrescentar mais duas atividades. Os adolescentes informaram se praticavam cada atividade (sim versus não), a frequência (dias/semana) e a duração (minutos/dia), em dias da semana anterior à coleta de dados. Foram consideradas apenas as atividades realizadas por pelo menos 10 minutos. Para fins de análise foram produzidos escores (minutos/semana) com base no somatório dos produtos da frequência versus duração de cada atividade, agrupando-as em: recreação (dança, andar de bicicleta e brincadeiras); esportes (basquetebol, handebol, voleibol, voleibol de praia, natação, futebol, futebol de praia, futsal, lutas, ginástica rítmica, skate, surfe, atletismo e tênis); exercício físico (ginástica aeróbica, musculação, caminhada e corrida); e deslocamento ativo (ir a pé/bicicleta para a escola e outros locais).

As variáveis sociodemográficas utilizadas neste estudo foram: sexo; idade em anos completos; escolaridade da mãe; turno de aula; cor da pele; classe econômica^24^; tempo de residência no bairro; e Índice de Massa Corporal (IMC) — com base nas medidas aferidas de massa corporal e estatura —, sendo o estado nutricional classificado seguindo os critérios da Organização Mundial da Saúde (OMS)^25^: baixo peso (<-2 desvios-padrão [dp]), peso normal (>-2 dp e <+1 dp), sobrepeso (>+1dp) e obesidade (>+2 dp).

Na descrição dos dados, foram empregados os procedimentos de estatística descritiva, como média, desvio-padrão e distribuição por frequência. Na comparação entre as características sociodemográficas e os tipos de prática entre os adolescentes excluídos e incluídos nas análises foram utilizados os testes χ^2^ e U de *Mann-whitney*, respectivamente. A regressão logística binária bruta foi utilizada para identificar se o padrão do *missing* era completamente aleatório, aleatório e/ou não aleatório^26^.

A Análise de Classe Latente foi utilizada para identificar os padrões de resposta aos itens (não=0 e sim=1) das características do ambiente mensuradas (Material suplementar 1). Para avaliar a qualidade de ajuste dos dados ao modelo e a definição do número de classes a serem escolhidas, foram adotados os seguintes critérios: menores valores de Critério de Informação de Akaike (AIC), Critério de Informação Bayesiano (BIC) e BIC ajustado; valores de entropia mais próximos de um; Teste de Razão de Probabilidade (LMR), *Bootstrap Likelihood Ratio Test* (BLRT) e Teste de Razão de Verossimilhança (TRV). Os testes utilizados na análise avaliam se o modelo com mais classes (*k* +1) é adequado para explicar os dados, comparativamente ao modelo com menos classes (*k*), sendo rejeitado o modelo com menos classes quando a significância estatística apresentar valor de p<0,05.

A regressão linear bruta e ajustada foi utilizada para analisar a relação dos padrões (variável independente) e os tipos de prática (variável dependente). Os fatores de confusão analisados foram: sexo (masculino=0 e feminino=1), idade (a partir da diferença entre a data de nascimento e a data de coleta de dados, e categorizada em: 10-11 anos=0 e 12-13 anos=1), classe econômica (A/B=0 e C/D/E=1), escolaridade da mãe (ensino: fundamental completo=0, médio incompleto=1 e médio completo ou mais=2), cor da pele (parda, preta, branca, amarela e indígena, e recategorizada em: branco=0 e não branco=1), turno de aula (manhã=0 e tarde=1), tempo de residência no bairro (<1 ano=0 e ≥1 ano=1) e estado nutricional (baixo peso/peso normal=0 e sobrepeso/obesidade=1). O método de seleção das variáveis no modelo ajustado foi *Forward,* permanecendo as que contribuíram para o melhor ajuste. A qualidade de ajuste do modelo foi avaliada com base no Fator de Inflação da Variância (VIF), sendo valores <5 considerados adequados para indicar a ausência de multicolinearidade; a normalidade dos resíduos foi avaliada por meio do teste de normalidade de Shapiro Wilk; e o Teste de Cook-Weisberg avaliou a homocedasticidade dos resíduos (na presença de heterocedasticidade, a correção robusta de White foi aplicada).

As análises descritivas e de associação foram realizadas no programa estatístico *Stata* 13.0 e a ACL, no *M-plus* 7.0. O nível de significância adotado foi de p<0,05.

### Disponibilidade de dados da pesquisa:

O conjunto de dados que dá suporte aos resultados deste estudo não está disponível publicamente.

## RESULTADOS

Dos 1.582 adolescentes convidados, 1.475 foram entrevistados. Após recusas e perdas (n=107), a amostra final foi composta de 1.066 adolescentes. Houve diferenças significativas entre os adolescentes incluídos e excluídos das análises para as variáveis sexo (p=0,004) e classe econômica (p=0,008) (Tabela 1). Os *missings* foram classificados como ao acaso (dados não apresentados em tabela).

**Tabela 1:** Comparação das características sociodemográficas, Índice de Massa Corporal (IMC) e atividade física entre os adolescentes incluídos e os excluídos das análises. João Pessoa, Paraíba, 2014.

Variáveis		Adolescentes incluídos nas análises (n=1.066)	Adolescentes excluídos nas análises (n=409)	Valor p†
		n (%)	n (%)	
Sexo				
	Masculino	477 (44,7)	217 (53,1)	0,004
	Feminino	589 (55,2)	192 (46,9)	
Idade (anos)				
	10 a 11	655 (61,4)	176 (59,3)	0,495
	12 a 13	411 (38,6)	121 (40,7)	
Cor da pele				
	Branco	211 (19,8)	73 (17,9)	0,410
	Não branco	853 (80,2)	334 (82,1)	
Turno de aula				
	Manhã	475 (44,6)	179 (43,7)	0,784
	Tarde	591 (55,4)	230 (56,2)	
Escolaridade da mãe				
	Fundamental incompleto	422 (39,6)	71 (47,9)	0,059
	Médio incompleto	303 (28,4)	43 (29,1)	
	Médio completo ou mais	341 (31,9)	34 (22,9)	
Classe econômica				
	A/B (mais alta)	452 (42,4)	68 (32,5)	0,008
	C/D/E (média e baixa)	614 (57,6)	141 (67,5)	
Estado nutricional				
	Baixo peso/peso normal	717 (67,3)	275 (69,6)	0,391
	Sobrepeso/obesidade	349 (32,7)	120 (30,4)	
		**Média (desvio-padrão)**	**Média (desvio-padrão)**	**Valor p** *
Atividade física (min/sem)				
	Recreação	168,2 (231,5)	158,6 (217,5)	0,153
	Esportes	233,4 (293,3)	228,7 (286,1)	0,353
	Exercício físico	42,6 (110,5)	42,1 (115,2)	0,121
	Deslocamento ativo	133,1 (134,5)	137,8 (150,4)	0,977

† Valor de p do teste χ^2^;

* valor de p do teste U de Mann-Whitney; min/sem=minutos por semana.

A maioria dos adolescentes era do sexo feminino (52,2%), tinha idade entre 10 e 11 anos (61,4%) e pertencia à classe econômica C/D/E (57,6%) (Tabela 1). Para as características percebidas do ambiente, houve uma elevada proporção de adolescentes que relataram a presença de "praças", com 77% (locais para AF); "Não ter medo de ficar em locais abertos", com 64,2% (segurança urbana); e "Segurança ao atravessar as ruas", com 76,4% (segurança no trânsito) (Figura 1).

**Figura 1:** Proporção de adolescentes que apresentaram percepção positiva sobre as características do ambiente para atividade física em João Pessoa, Paraíba, em 2014.

1: academias de ginástica ou de lutas; 2: quadras de esportes não cobertas; 3: ginásio poliesportivo coberto; 4: pistas/locais para caminha/corrida; 5: praças; 6: parques/áreas verdes; 7: parquinhos (playgrounds); 8: pistas de skate/patins; 9: não ter medo de ficar em locais abertos; 10: não ter medo de ficar em locais abertos com os amigos; 11: não ter medo de andar nas ruas (dia); 12: não ter medo de andar nas ruas (noite); 13: não ter trânsito intenso de carros/motos; 14: não ter motorista dirigindo em alta velocidade; 15: segurança ao atravessar as ruas; 16: não existir muitos roubos/assaltos.

Foram avaliados cinco modelos de forma sequencial (*k + 1* classe) e, de acordo com os indicadores da qualidade de ajuste, o melhor modelo identificado na análise foi o de quatro classes latentes (Tabela 2). As classes identificadas foram denominadas como: "não diversificado e inseguro" (classe 1, n=277 — 26%), "não diversificado e seguro" (classe 2, n=145 — 13,6%), "diversificado e inseguro" (classe 3, n=328 — 30,8%) e "diversificado e seguro" (classe 4, n=316 – 29,6%) (Figura 2).

**Figura 2:** Classes latentes dos padrões das características do ambiente para atividade física em adolescentes. João Pessoa, Paraíba, 2014.

Classe 1: n=277 (26%) — não diversificado-inseguro; Classe 2: n=145 (13,6%) — não diversificado-seguro; Classe 3: n=328 (30,8%) — diversificado-inseguro e; Classe 4: n=316 (29,6%) — diversificado-seguro; Eixo x: representa os itens que compõem a escala que mensura as características do ambiente; Eixo y: representa a probabilidade de o adolescente ter uma percepção positiva (sim) para a presença do item no bairro; 1: academias de ginástica ou de lutas; 2: quadras de esportes não cobertas; 3: ginásio poliesportivo coberto; 4: pistas/locais para caminha/corrida; 5: praças; 6: parques/áreas verdes; 7: parquinhos (playgrounds); 8: pistas de skate/patins; 9: não ter medo de ficar em locais abertos; 10: não ter medo de ficar em locais abertos com os amigos; 11: não ter medo de andar nas ruas (dia); 12: não ter medo de andar nas ruas (noite); 13: não ter trânsito intenso de carros/motos; 14: não ter motorista dirigindo em alta velocidade; 15: segurança ao atravessar as ruas; 16: não existir muitos roubos/assaltos.

**Tabela 2:** Parâmetros da qualidade de ajuste do modelo para a formação das classes latentes a partir das características do ambiente para atividade física em adolescentes de João Pessoa, Paraíba, em 2014.

Parâmetros do modelo		Classes latentes					
N.° de classes latentes			1	2	3	4	5
Log-Likelihood			-10.906,4	-10.450,1	-10.151,7	-10.032,2	-9.982,3
AIC^a^			21.844,7	20.966,9	20.403,5	20.198,4	20.132,7
BIC^a^			21.924,2	21.131,0	20.652,1	20.531,5	20.550,3
BIC - Ajustado			21.873,4	21.526,1	20.493,3	20.318,7	20.283,5
VLMR (valor p)			-	-10.906 (<0,001)	-10.450,4 (0,003)	-10.151,7 (<0,001)	-10.032,2 (0,376)
LMR (valor p)			-	904,1 (<0,001)	592,4 (0,003)	237,1 (<0,001)	98,8 (0,379)
BRLT (valor p)			-	-10.906,4 (<0,001)	-10.450,5 (<0,001)	-10.151,7 (<0,001)	-10.032,2 (<0,001)
Entropia^b^			1,000	0,738	0,736	0,737	0,721
Classes		n (%)	Probabilidades				
			1	2	3	4	5
1 classe1		1.066 (100)	1,000				
2 classes							
	classe 1	645 (60,4)	0,924	0,076			
	classe 2	421 (39,5)	0,079	0,921			
3 classes							
	classe 1	331 (31)	0,866	0,086	0,048		
	classe 2	380 (35,6)	0,065	0,881	0,054		
	classe 3	355 (33,2)	0,053	0,053	0,894		
4 classes							
	classe 1	145 (13,6)	0,865	0,070	0,064	0,002	
	classe 2	277 (26,0)	0,048	0,858	0,019	0,074	
	classe 3	315 (29,6)	0,039	0,027	0,839	0,095	
	classe 4	328 (30,8)	0,001	0,069	0,072	0,858	
5 classes							
	classe 1	267 (25,1)	0,829	0,036	0,076	0,046	0,013
	classe 2	141 (13,2)	0,072	0,861	0,003	0,002	0,062
	classe 3	167 (15,6)	0,108	0,008	0,736	0,079	0,068
	classe 4	228 (21,4)	0,034	0,001	0,077	0,832	0,057
	classe 5	263 (24,7)	0,012	0,053	0,039	0,054	0,841

AIC: *Akaike Information Criterion*; BIC: *Bayesian Information Criterion*; VLMR: *Vuong-Lo-Mendell-Rubin Likelihood Ratio Test*; LMR: *Lo-Mendell-Rubin Adjusted*; BRLT: *Bootstrap Likelihood Ratio Test*;

a Valores menores indicam melhor ajuste do modelo;

b Medida da incerteza de classificação limitada entre 0 e 1, na qual valores maiores refletem melhor ajuste do modelo.

Na análise bruta, houve associações positivas e significativas entre perceber o ambiente "diversificado e seguro" e as atividades recreativas (β=42,08; IC95% 5,09–71,64) e de esportes (β=63,07; IC95% 16,27–109,87). Na análise ajustada (Tabela 3), os esportes perderam significância estatística, e perceber o ambiente do bairro como "diversificado e seguro" continuou associado às atividades recreativas (β=42,16; IC95% 4,35–79,97).

**Tabela 3:** Regressão linear bruta e ajustada para a associação entre os padrões das características percebidas do ambiente do bairro e os tipos de prática em adolescentes de João Pessoa, Paraíba, em 2014.

Variáveis	Tipos de prática (minutos/semana)							
	Recreação		Esportes		Exercício físico		Deslocamento ativo	
	Modelo bruto	Modelo ajustado	Modelo bruto	Modelo ajustado	Modelo bruto	Modelo ajustado	Modelo bruto	Modelo ajustado
	β (IC95%)	β (IC95%)	β (IC95%)	β (IC95%)	β (IC95%)	β (IC95%)	β (IC95%)	β (IC95%)
Classe 1	1	1	1	1	1	1	1	1
Classe 2	43,41 (-4,02–90,86)	41,10 (-7,21–89,42)	40,65 (-19,36–100,67)	-25,89 (-82,99–31,19)	6,66 (-16,02–29,35)	3,68 (-19,27–26,63)	5,41 (-22,14–32,97)	4,99 (-22,87–32,85)
Classe 3	34,38 (-2,86–71,63)	33,90 (-3,37–71,18)	12,48 (-34,64–59,61)	14,34 (-29,70–58,40)	10,07 (-7,73–27,89)	9,52 (-8,18–27,24)	18,34 (-3,29–39,98)	17,73 (-3,76–39,24)
Classe 4	42,08 (5,09–71,64)	42,16 (4,35–79,97)	63,07 (16,27–109,87)	7,24 (-37,43–51,92)	10,69 (-6,99–28,38)	9,66 (-8,30–27,62)	-5,23 (-26,72–16,25)	-1,84 (-23,65–19,95)

β: coeficiente de beta; IC95%: intervalo de confiança de 95%; Modelo ajustado por sexo, idade, classe econômica, escolaridade da mãe, cor da pele, estado nutricional e turno de estudo; Classe 1: "não diversificado e inseguro"; Classe 2: "não diversificado e seguro"; Classe 3: "diversificado e inseguro"; Classe 4: "diversificado e seguro". O valor de VIF em todos modelos foi <2, o que indica a ausência de multicolinearidade; o Teste de Cook-Weisberg apresentou valores de p<0,001, indicando a presença de heterocedasticidade, e a correção robusta de White foi aplicada.

## DISCUSSÃO

Os resultados do presente estudo identificaram a formação de quatro padrões distintos para as características percebidas do ambiente ("não diversificado e inseguro", "não diversificado e seguro", "diversificado e inseguro" e "diversificado e seguro") em relação à prática de AF dos adolescentes. Desses, o padrão "diversificado e seguro" esteve associado às atividades recreativas dos adolescentes.

A hipótese de que os adolescentes percebem as características do ambiente de forma heterogênea — principalmente quando diferentes domínios como a disponibilidade de locais para AF e a segurança urbana e no trânsito são combinados — foi confirmada. Isso reflete como o ambiente foi percebido e interpretado pelos adolescentes em seu contexto de vida real. Estudos realizados no Brasil identificaram a formação de até quatro padrões distintos de ambiente considerando também a percepção dos adolescentes sobre a disponibilidade de locais para AF e de aspectos relacionados à segurança^19,20^.

Por outro lado, apenas Castro et al.^19^ investigaram os padrões ("vizinhança urbanizada e favorável à AF", "vizinhança segura e sociável" e "insegura e pouco sociável") com base nas respostas fornecidas de forma individual a cada característica do ambiente mensurada. Dos padrões identificados, a "vizinhança urbanizada e favorável à AF" esteve associada a maiores chances (OR=1,72; IC95% 1,29–2,29) de realizar AF no lazer. Destaca-se que os participantes deste estudo eram adolescentes mais velhos e adultos^19^, que em geral têm maior autonomia para se deslocar no bairro e preferências específicas por determinados tipos de prática, diferentemente de adolescentes mais jovens, que tendem a ser mais dependentes dos pais e ter preferências por outros tipos de prática.

No presente estudo, os adolescentes que percebiam o bairro em que residiam como "diversificado e seguro" apresentavam maior tempo de prática de atividades do tipo recreativas quando comparados aos que percebiam o ambiente "não diversificado e inseguro". É possível que essa relação encontrada esteja relacionada ao perfil de um grupo de adolescentes cujos pais e/ou responsáveis tenham maior condição socioeconômica e/ou conhecimento sobre a prática da AF. Assim, podem residir em bairros que disponham de locais, equipamentos recreacionais, e segurança urbana e no trânsito, oportunizando aos adolescentes maior tempo nesse tipo de prática.

Outra explicação para esse achado pode estar nas características inerentes desse tipo de prática (por exemplo, andar de bicicleta e brincadeiras), que em geral não exige planejamento prévio, horário fixo, regras, normas, estrutura física e/ou equipamentos específicos para a realização. Portanto, bairros com maior variabilidade de locais para AF e baixos índices de criminalidade tornam-se mais atrativos para a prática de atividades recreativas, pois favorecem encontros entre adolescentes durante seus momentos de lazer.

A ausência de associação entre os padrões identificados com o exercício físico e o esporte neste estudo pode ter acontecido devido às características e demandas dessas AFs, que em geral necessitam de locais, espaços, equipamentos e materiais específicos. Embora estudos anteriores com adolescentes em faixa etária similar tenham identificado uma associação positiva entre as características do ambiente e a prática de exercícios físicos^27^ e esportes^7^, isso não foi observado no presente estudo. É possível que essa ausência de associação possa estar relacionada ao questionário utilizado para mensurar a AF, que não identificava o local onde os adolescentes realizavam as práticas relatadas.

Embora os exercícios físicos possam ser realizados em diversos espaços, como ao ar livre ou em academias de ginástica/lutas, eles geralmente requerem instruções sobre como praticá-los e o acesso a equipamentos específicos. Isso pode representar uma barreira para adolescentes de menor condição socioeconômica, já que a maioria dos adolescentes que participaram deste estudo (57,6%) pertenciam à classe econômica média/baixa. No que diz respeito a ausência de associação entre os padrões e as atividades esportivas, o ambiente escolar e as aulas de educação física podem ter um papel mais relevante para aqueles que não têm locais disponíveis no bairro, materiais e equipamentos necessários. Além disso, as escolas podem ser os espaços mais utilizados para essas práticas, oferecendo a companhia dos colegas e a possibilidade de participar de modalidades como voleibol, futsal, entre outros esportes coletivos.

Neste estudo também não foram identificadas associações entre os padrões do ambiente e o deslocamento ativo. É possível que isso tenha ocorrido porque esse tipo de atividade pode ter sido realizada, em grande parte, por necessidade e não pela escolha do adolescente, devido à ausência de uma outra opção de transporte para se deslocarem entre os trajetos de casa para a escola, casa dos amigos ou outros lugares^28^. Estudos demonstraram que o deslocamento ativo realizado no trajeto casa-escola tem sido associado a baixa renda familiar^29^, frequentar escola pública^30^ e falta de um transporte em casa — como o automóvel^31^.

Embora tenha sido utilizada uma escala validada para mensurar as características do ambiente^22^, é possível que, para os adolescentes que participaram deste estudo, existam outras características que não foram mensuradas, as quais podem ser mais preditoras para os tipos de prática de AF analisados. Estudos que utilizaram escalas curtas de ambiente^19^ não encontraram diversidade nas associações entre os padrões identificados e a AF quando comparados aos que utilizaram escalas com uma maior quantidade de domínios e itens^20^. No entanto, esses estudos mensuraram a AF total, no domínio do lazer^19^ ou por intensidade (leve, moderada, vigorosa) de prática^20^. Isso pode não refletir o interesse e as preferências dos adolescentes pelos tipos de prática, limitando a uma avaliação de como a percepção a um conjunto de características do ambiente pode estar relacionada de forma específica aos tipos de prática.

Os achados do presente estudo demonstraram a possibilidade da adoção de uma outra forma de analisar as características do ambiente e que as percepções a determinados atributos quando combinados se relacionam de forma específica com o tipo de AF mensurado. Isso pode auxiliar no desenvolvimento de intervenções cujas ações estão relacionadas à mudança, seja no ambiente físico dos bairros, seja na percepção dos adolescentes sobre os locais onde residem. Desse modo, sugere-se que estudos futuros com o objetivo de investigar os padrões do ambiente para atividade física possam adicionar outras características do ambiente para avaliar a sua relação com os diferentes tipos de prática de AF dos adolescentes.

Este estudo apresenta algumas limitações. O questionário utilizado para mensurar a AF dos adolescentes não incluía perguntas sobre o local onde as atividades eram realizadas (por exemplo, na escola, no bairro). Dado que os participantes pertenciam, em sua maioria, à classe econômica média/baixa, é possível que muitas das AFs relatadas tenham sido praticadas na escola e/ou nas aulas de educação física. Outra limitação está relacionada à escala de ambiente, pois é possível que a baixa quantidade de itens pode não ter capturado características do bairro que poderiam ser mais relevantes para os tipos de AF analisados, o que pode ter implicado na quantidade de associações identificadas.

Apesar das limitações, o estudo utilizou instrumentos validados para mensurar as características do ambiente^22^ e a AF^23^, aplicados por uma equipe treinada e seguindo um protocolo para a coleta de dados; e uma análise estatística robusta, que permitiu identificar possíveis combinações entre atributos de diferentes domínios do ambiente percebido e relacioná-las com os tipos de prática de AF dos adolescentes.

Conclui-se que as características dos diferentes domínios do ambiente, como a disponibilidade de locais para AF e a segurança urbana e no trânsito, estão inter-relacionadas e que, ao serem combinadas, os adolescentes apresentaram padrões de percepções heterogêneos, demonstrando que o bairro pode não ter a mesma representatividade para todos. Adolescentes que percebiam o ambiente do bairro como diversificado e seguro apresentaram maior tempo de prática nas atividades do tipo recreativas.

## Material Suplementar

Material Suplementar 1
